# Evaluating the safety and efficiency of robotic dispensing systems

**DOI:** 10.1186/s40780-022-00255-w

**Published:** 2022-10-01

**Authors:** Tomoki Takase, Norio Masumoto, Naoki Shibatani, Yusaku Matsuoka, Fumiaki Tanaka, Masaki Hirabatake, Hiroko Kashiwagi, Itaru Nishioka, Hiroaki Ikesue, Tohru Hashida, Naoshi Koide, Nobuyuki Muroi

**Affiliations:** 1 Department of Pharmacy, Kobe City Medical Center General Hospital, 2-1-1, Minatojima Minamimachi, Chuo-ku, Kobe, Hyogo 650-0047 Japan; 2 Deloitte Analytics, Deloitte Touche Tohmatsu LLC, 3-2-3, Marunouchi, Chiyoda-ku, Tokyo, 100-8360 Japan; 3 Social Solution Initiative, Osaka University, 2-8, Yamadaoka, Suita, Osaka, 565-0871 Japan; 4 Research Center On Ethical, Legal and Social Issues, Osaka University, 2-8, Yamadaoka, Suita, Osaka, 565-0871 Japan

**Keywords:** Pharmacist, Robot, Dispensing device, Dispensing error, Dispensing time

## Abstract

**Background:**

Although automated dispensing robots have been implemented for medication dispensing in Japan, their effect is yet to be fully investigated. In this study, we evaluated the effect of automated dispensing robots and collaborative work with pharmacy support staff on medication dispensing.

**Methods:**

A robotic dispensing system integrating the following three components was established: (1) automated dispensing robot (Drug Station®), which is operated by pharmacy support staff, (2) automated dispensing robot for powdered medicine (Mini DimeRo®), and (3) bar-coded medication dispensing support system with personal digital assistance (Hp-PORIMS®). Subsequently, we evaluated the incidences of dispensing errors and dispensing times before and after introducing the robotic dispensing system. Dispensing errors were classified into two categories, namely prevented dispensing errors and unprevented dispensing errors. The incidence of dispensing errors was calculated as follows: incidence of dispensing errors = total number of dispensing errors/total number of medication orders in each prescription.

**Results:**

After introducing the robotic dispensing system, the total incidence of prevented dispensing errors was significantly reduced (0.204% [324/158,548] to 0.044% [50/114,111], *p* < 0.001). The total incidence of unprevented dispensing errors was significantly reduced (0.015% [24/158,548] to 0.002% [2/114,111], *p* < 0.001). The number of cases of wrong strength and wrong drug, which can seriously impact a patient’s health, reduced to almost zero. The median dispensing time of pharmacists per prescription was significantly reduced (from 60 to 23 s, *p* < 0.001).

**Conclusions:**

The robotic dispensing system enabled the process of medication dispensing by pharmacist to be partially and safely shared with automated dispensing robots and pharmacy support staff. Therefore, clinical care for patients by pharmacists could be enhanced by ensuring quality and safety of medication.

**Supplementary Information:**

The online version contains supplementary material available at 10.1186/s40780-022-00255-w.

## Background

Healthcare systems are rapidly shifting from a single hospital-based care module to a community-based regional collaborative care system. Pharmacists can contribute toward maximizing patient safety and efficacy of pharmacotherapy, from hospital to community care. In April 2019, Japanese Ministry of Health, Labour, and Welfare released a notification titled “the conception of medication dispensing” to pharmacists to ensure sufficient time for clinical care of patients [1]. This notification describes that preparing medicines is one of delegable works from pharmacists to pharmacy support staff. In most hospitals, dispensing system/process includes verifying the appropriateness of the prescription, such as the dose, in individual disorders and drug–drug interactions as well as manually selecting medicines from shelves. Because dispensing and verification processes are complex, preventing human errors is difficult. Thus, dispensing errors can inevitably occur at a certain rate [2, 3]. However, incidents because of dispensing errors can cause iatrogenic harm in patients. Therefore, minimizing human errors in drug dispensing is essential. Because highly skilled pharmacists are required to prevent human errors in manual medication dispensing, delegating even part of the dispensing process (preparing prescribed medicines) from pharmacists to pharmacy support staff is difficult.

Recently, automated dispensing robots have been implemented in Japan. They have achieved remarkable reduction in dispensing errors and improved the efficiency of dispensing processes [2, 4, 5]. The ability of robots to provide fast and accurate dispensing allows pharmacists to spend more time on clinical care for patients, thereby adding value to their clinical role [2, 4, 5]. Despite several potential advantages of integrating automated dispensing robots and collaboration with pharmacy support staff, the safety and the efficiency of such systems is yet to be fully evaluated in Japan.

We established the “robotic dispensing system” with the following three components: (1) automated dispensing robot operated by pharmacy support staff, (2) automated dispensing robot for powdered medicine, and (3) bar-coded medication dispensing support system with personal digital assistance (PDA). Notably, pharmacy support staff engaged in preparing prescribed medicines using the automated dispensing robot in the robotic dispensing system. There is no independent organization of pharmacy technicians in Japan. Therefore, we trained pharmacy support staff to collaborate with pharmacists for medication dispensing.

We investigated reduction in dispensing errors and dispensing time before and after introducing the robotic dispensing system comprising collaborative working model with pharmacists and pharmacy support staff.

## Methods

### Study site

Kobe City Medical Center General Hospital is a 768-bed acute phase hospital in Japan. An average of 500 prescriptions including single or multiple medication orders per prescription are handled each day in the hospital pharmacy. We analyzed proportions of prescriptions using each dispensing device in the study periods, as described later.

### Robotic dispensing system

#### Newly implemented dispensing devices

**Fig. 1:**
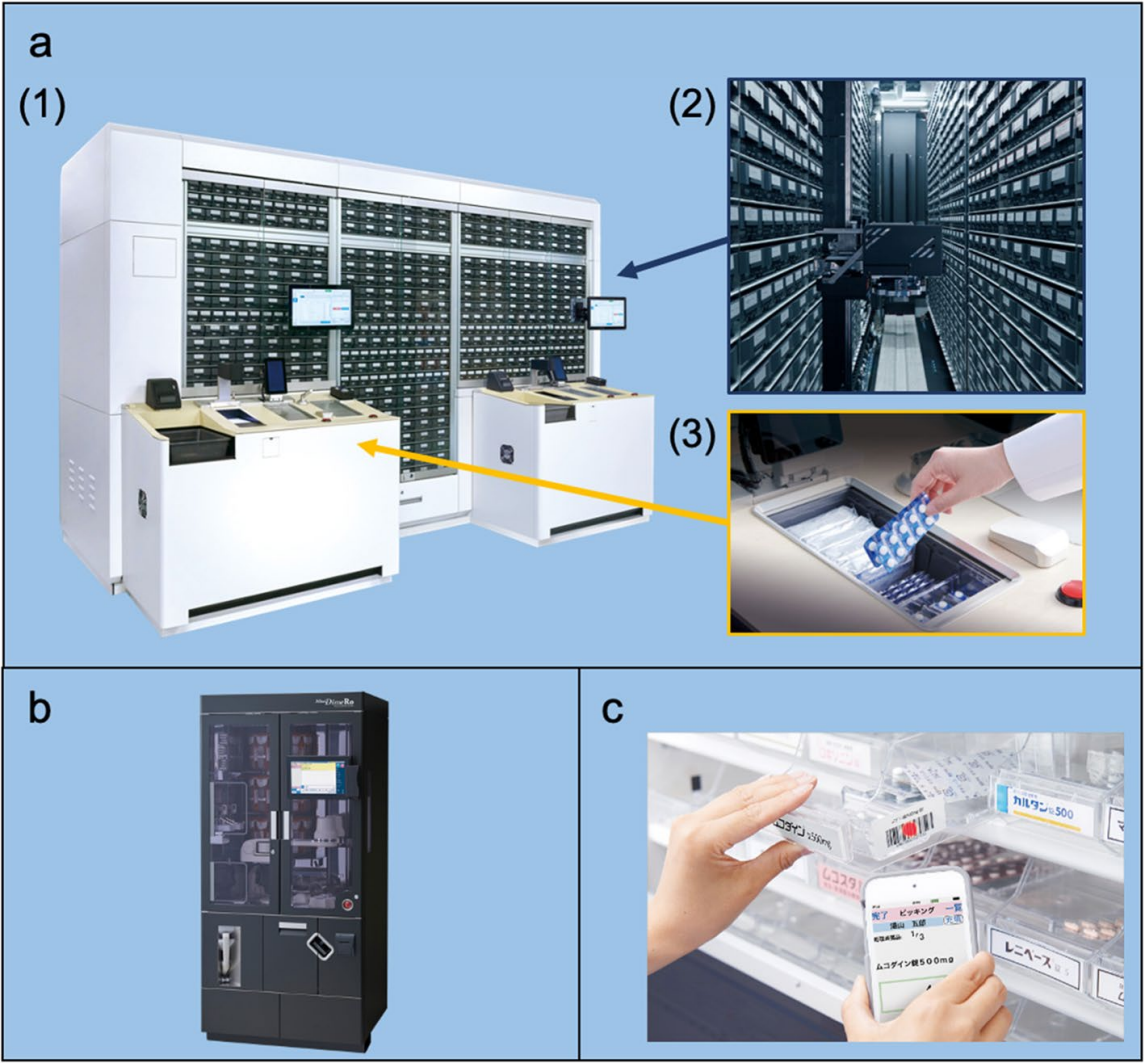
Newly implemented dispensing devices. **a** Automated dispensing robot (Drug Station®), (1) outside view, (2) storage bins and robotic arms, (3) slot. **b** Automated dispensing robot for powdered medicine (Mini DimeRo®). **c** Bar-coded medication dispensing support system with using PDA (Hp-PORIMS.®)

We introduced the robotic dispensing system integrating the following three components (Fig. 1) in February 24, 2021.

- *Automated dispensing robot*
  We implemented an automated dispensing robot (Drug Station®, Yuyama Co., Ltd., Osaka, Japan) which stores a maximum of 1,200 single unit packages of oral medicines such as tablets, capsules, powders, liquids, and topical medications. This robot is linked to our hospital computerized physician order entry (CPOE) system (HOPE/EGMAIN-GX®, Fujitsu, Ltd., Tokyo, Japan). Pharmacists or pharmacy support staff pick up the ordered quantity of medicines according to the instructions on the screen from the storage bins, which automatically moves to the handling slots of Drug Station® by ordered prescription data in the CPOE system. After the medicines pick up by a pharmacist or a pharmacy support staff, the number of medicines is graphically confirmed by using the built-in camera, and/or their weight are confirmed by the built-in electronic scale. Because of these reliable functions, this robot can support preparing medications accurately for both pharmacists and pharmacy support staff.
  In our hospital formulary, a total of 749 oral or topical medicines were approved by the Pharmacy and Therapeutic Committee (P&T Committee). Among them, 623 (83.2%) were stored in the automated dispensing robot. The remaining 126 medicines could not be stored in the robot because they require low-temperature storage; strict legal controls, such as opioid analgesics; or are packed in large packaging that could not fit in the storage bin.
- *Automated dispensing robot for powdered medicine*
  We also implemented an automated dispensing robot for powdered medicine (Mini DimeRo®, Yuyama Co., Ltd., Osaka, Japan), which was linked to our hospital CPOE system. After physicians ordered powdered medicines, this robot automatically weighed and packed them. This system can prepare powdered medicines precisely, and the time required to prepare powdered medicines was considerably shorter than that in using conventional automatic packaging machines [4]. In our hospital, among the 71 powdered medicines approved by the P&T Committee, 42 (59.2%) were stocked into the cassettes of the robot.
- *Bar-coded medication dispensing support system*

We implemented a bar-coded medication dispensing support system with PDA (Hp-PORIMS®, Yuyama Co., Ltd., Osaka, Japan), which was connected to our hospital CPOE system. This system control prescriptions and medicine packages by bar coding and collates using PDA whether the drug is picked correctly according to the prescription. This system considerably reduced dispensing errors [6]. This system was used for 126 oral or topical medicines, which could not be stored in the automated dispensing robot. Additionally, 60 self-injectable drugs, approved by the P&T Committee, were also dispensed using this system.

#### Role of pharmacy support staff

Pharmacy support staff engaged in preparing prescribed medicines using the automated dispensing robot. Pharmacy support staff worked from 8:45 to 17:30 on weekdays. Other than those time, pharmacists executed all flows of medication dispensing steps.

#### Operation flow before and after introducing the robotic dispensing system

Before introducing the robotic dispensing system (until February 23, 2021), pharmacists verified each prescription and manually prepared and dispensed medicines. Dispensed medicines and the prescriptions were subsequently verified by another pharmacist. After introducing the robotic dispensing system (since February 24, 2021), pharmacists verified each prescription, and pharmacists or pharmacy support staff prepared medicines using the automated dispensing robot. With the exception of medicines stored in the automated dispensing robot, medicines were collated using bar-coded medication dispensing support system with PDA. Subsequently, the prepared and dispensed medicines, and their prescriptions were verified by another pharmacist. Pharmacists trained pharmacy support staff on the operation of the robotic dispensing system for 2 weeks. Pharmacy support staff then started operating independently since June 1, 2021.

**Fig. 2:**
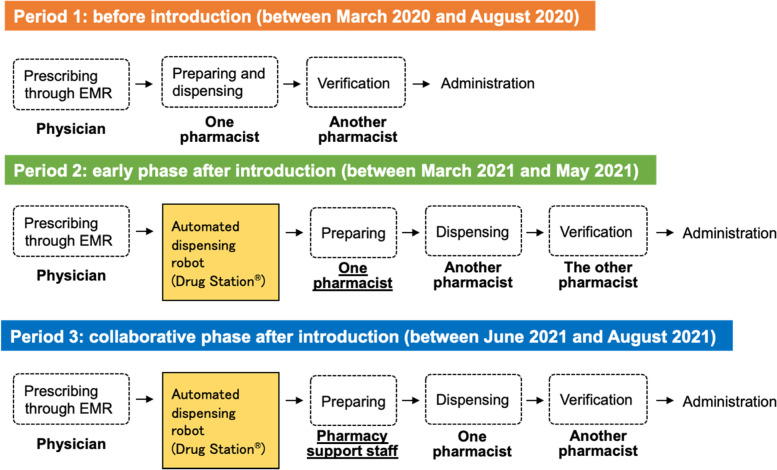
Dispensing process flowchart before and after introducing the robotic dispensing system in the study period. Before introducing the robotic dispensing system (period 1), pharmacists manually prepared and dispensed medicines. After introducing the robotic dispensing system (period 2, 3), pharmacists or pharmacy support staff prepared medicines using automated dispensing robot. EMR: electronic medical records

We defined the study periods as follows (Fig. 2): period 1 (before introduction: between March 2020 and August 2020), period 2 (early phase after introduction: between March 2021 and May 2021), and period 3 (collaborative phase after introduction: between June 2021 and August 2021) to evaluate the after-mentioned incidences of dispensing errors and dispensing time.

### Incidences of dispensing errors

We classified dispensing errors into two categories, namely prevented dispensing errors and unprevented dispensing errors [7]. The prevented dispensing errors denoted errors detected by pharmacists before the medicines provided from the pharmacy to clinical wards or outpatients. By contrast, unprevented dispensing errors denoted errors that were detected by other medical staff or patients after the medicines provided from the pharmacy to clinical wards or to outpatients. Each incident was recorded when pharmacists, other medical staff, or patients detected the errors. The incidences of prevented and unprevented dispensing errors were compared before and after introducing the robotic dispensing system: periods 1, 2, and 3 (Fig. 2). The participating pharmacists numbered 59, 60, and 60, respectively, in this study, on dispensing errors for periods 1, 2 and 3, respectively.

The incidence of dispensing errors was calculated as follows:

incidence of dispensing errors = total number of dispensing errors/total number of medication orders in each prescription.

Types of dispensing errors were defined as follows [8–10]: wrong drug (e.g., caused by similar name: “Norvasc® tablet” and “Nolvadex® tablet”), wrong quantity (caused by miscount), wrong strength (caused by selection error: e.g., “bisoprolol tablet 0.625 mg” and “bisoprolol tablet 2.5 mg”), wrong dosage form (e.g., “diclofenac suppository” and “diclofenac tablet”), and others.

### Dispensing time

We randomly selected the prescriptions (i.e., each pharmacist picked up the prescriptions without looking at the contents) in period 1, 2, and 3 (Fig. 2), and compared the time spent on dispensing per prescription in each period. Additionally, the dispensing time was classified into three categories as follows: (a) work time of pharmacists, (b) work time of robot, and (c) work time of pharmacy support staff. This study involved all types of prescriptions regardless of the use of Drug Station®. A total of 10 pharmacists with 1–42 years of experience (one year, *n* = 6; four years, *n* = 1; eleven years, *n* = 1; twenty-six years, *n* = 1; forty-two years, *n* = 1) and 5 pharmacy support staff participated in the study. Pharmacy support staff took turns in operating the automated dispensing robot. The work time of robot was defined as the operating time of Drug Station® for each prescription. We defined the work time of robot, considering only the operating time of Drug Station®, because Mini DimeRo® automatically weighs and packs powdered medicines without human intervention unlike Drug Station®. In periods 2 and 3, the dispensing times of powdered medicines and using Hp-PORIMS® were included into the work time of pharmacists. In period 1, the work time of pharmacists (defined as the time required for manually preparing and dispensing medicines by pharmacist) was measured. In period 2, the work time of robot and pharmacists (including the total time required for preparing medicines using Drug Station® by one pharmacist and dispensing medicines by another pharmacist) were measured. In period 3, the work time of robot, and the work time of pharmacy support staff (defined as the time required for preparing medicines using Drug Station® by pharmacy support staff), and the work time of pharmacists (defined as the time required for dispensing medicines by pharmacist) were measured.

### Statistical analysis

Categorical data were presented as numbers (percentage) and were compared between groups using Fisher’s exact test. Continuous data are presented as medians (interquartile ranges), and the Mann–Whitney *U* test was used to compare the groups. All statistical analyses were performed using JMP 14.2.0 (SAS Institute Inc., Cary, NC, USA). To compare the incidences of dispensing errors and dispensing time among the study periods, the Bonferroni corrections were applied to determine the level of significance for each group (*p* < 0.0167).

## Results

### Proportions of prescriptions using the robotic dispensing system among the study periods

Proportions of prescriptions using the robotic dispensing system in each study period are displayed in Table 1. The number of prescriptions dispensed were 77,199, 51,482, and 54,822, the total number of medication orders per prescription dispensed were 158,548, 106,611 and 114,111 in periods 1, 2, and 3, respectively. In period 2, the number of drugs using the automated dispensing robot, automated dispensing robot for powdered medicine, and bar-coded medication dispensing support system were 81,073 (76.0%), 4,380 (4.1%), and 9,252 (8.7%), respectively. In period 3, proportions were 87,742 (76.9%), 4,091 (3.6%), and 9,975 (8.7%), respectively. The dispensing device use rates were similar in periods 2 and 3.

**Table 1:** Proportions of prescriptions using the robotic dispensing system

	**Period 1**	**Period 2**	**Period 3**
Number of prescriptions dispensed, n	77,199	51,482	54,822
Total number of medication orders per prescription dispensed, n (%)	158,548 (100%)	106,611 (100%)	114,111 (100%)
Number of medication orders using dispensing devices, n (%)			
Drug Station®	0 (0%)	81,073 (76.0%)	87,742 (76.9%)
Mini DimeRo®	0 (0%)	4,380 (4.1%)	4,091 (3.6%)
Hp-PORIMS®	0 (0%)	9,252 (8.7%)	9,975 (8.7%)
Not used above 3 dispensing devices	158,548 (100%)	11,907 (11.2%)	12,303 (10.8%)

Proportions of prescriptions before and after introducing the robotic dispensing system among three periods of trial are displayed. Period 1 (between March 2020 and August 2020) is the time before introducing the robotic dispensing system, period 2 (between March 2021 and May 2021) indicates early phase after introducing the robotic dispensing system, and period 3 (between June 2021 and August 2021) indicates the collaborative phase after introducing the robotic dispensing system

### Effects of the robotic dispensing system reducing dispensing errors

The dispensing errors related to oral or topical medicines, and self-injectable drugs were detected in the study periods. The incidences of prevented dispensing errors by error type were wrong quantity (0.107% vs 0.026% vs 0.028%), wrong strength (0.052% vs 0.003% vs 0%), wrong drug (0.025% vs 0.005% vs 0.001%), wrong dosage form (0.010% vs 0% vs 0%), others (0.010% vs 0.021% vs 0.015%), and total (0.204% vs 0.054% vs 0.044%) in periods 1, 2, and 3, respectively (Table 2). Among them, wrong quantity, wrong strength, wrong drug, wrong dosage form and total were significantly reduced in periods 2 and 3 compared with those in period 1 (all *p* < 0.001). No significant difference was observed in all types of the incidences of prevented dispensing errors between periods 2 and 3.

**Table 2:** Incidence of dispensing errors before and after introducing the robotic dispensing system

	**Period 1**	**Period 2**		**Period 3**		
	**Number (%)**	**Number (%)**	***p*-values vs Period 1**	**Number (%)**	***p*-values vs Period 1**	***p*-values vs Period 2**
Prescribed medications	158,548 (100%)	106,611 (100%)		114,111 (100%)		
Prevented dispensing errors						
Wrong quantity	170 (0.107%)	28 (0.026%)	< 0.001*	32 (0.028%)	< 0.001*	0.897
Wrong strength	83 (0.052%)	3 (0.003%)	< 0.001*	0 (0%)	< 0.001*	0.113
Wrong drug	39 (0.025%)	5 (0.005%)	< 0.001*	1 (0.001%)	< 0.001*	0.113
Wrong dosage form	16 (0.010%)	0 (0%)	< 0.001*	0 (0%)	< 0.001*	NA
Others	16 (0.010%)	22 (0.021%)	0.031	17 (0.015%)	0.292	0.339
TOTAL	324 (0.204%)	58 (0.054%)	< 0.001*	50 (0.044%)	< 0.001*	0.290
Unprevented dispensing errors						
Wrong quantity	12 (0.008%)	3 (0.003%)	0.123	0 (0%)	0.002*	0.113
Wrong strength	1 (0.001%)	0 (0%)	1.000	0 (0%)	1.000	NA
Wrong drug	4 (0.003%)	0 (0%)	0.154	0 (0%)	0.145	NA
Wrong dosage form	2 (0.001%)	0 (0%)	0.519	0 (0%)	0.513	NA
Others	5 (0.003%)	2 (0.002%)	0.709	2 (0.002%)	0.707	1.000
TOTAL	24 (0.015%)	5 (0.005%)	0.013*	2 (0.002%)	< 0.001*	0.274

The prevented dispensing errors were detected by pharmacists before the medicines were provided from the pharmacy to clinical wards or to outpatients. By contrast, unprevented dispensing errors indicates errors detected by other medical staff or patients after the medicines were provided from the pharmacy to clinical wards or to outpatients. The incidences of prevented and unprevented dispensing errors among three periods of trial are displayed. Period 1 (between March 2020 and August 2020) is the period before introducing the robotic dispensing system, period 2 (between March 2021 and May 2021) indicates the early phase after introducing the robotic dispensing system, and period 3 (between June 2021 and August 2021) indicates the collaborative phase after introducing the robotic dispensing system. The incidences were presented as the number of errors divided by the numbers of total medication orders in each prescription

*Abbreviation*: *NA* Not applicable

^*^ Statistically significant after adjustment using the Bonferroni correction (*p* < 0.0167 for Fisher's exact test)

The incidences of unprevented dispensing errors by error type were wrong quantity (0.008% vs 0.003% vs 0%), wrong strength (0.001% vs 0% vs 0%), wrong drug (0.003% vs 0% vs 0%), wrong dosage form (0.001% vs 0% vs 0%), others (0.003% vs 0.002% vs 0.002%), and total (0.015% vs 0.005% vs 0.002%) in periods 1, 2, and 3, respectively (Table 2). Among them, wrong quantity was significantly reduced in period 3 compared with that in period 1 (*p* = 0.002). Total unprevented dispensing errors were significantly reduced in periods 2 and 3 compared with those in period 1 (*p* = 0.013 and *p* < 0.001, respectively). No significant difference was observed in all types of the incidences of unprevented dispensing errors between periods 2 and 3.

### Effects of the robotic dispensing system reducing the dispensing time per prescription

The characteristics of prescription used for evaluating the dispensing time are presented in Supplementary Table 1. The number of prescriptions for evaluating dispensing time were 223, 184, and 310 in periods 1, 2, and 3, respectively. The work time of pharmacists, robot, and pharmacy support staff in each period are presented in Table 3. The work time of pharmacists in period 3 (median of 23 s) was significantly lower than that in periods 1 (median of 60 s) or 2 (median of 69 s) (both *p* < 0.001). Although the total dispensing time significantly increased from periods 1 (median of 60 s) to 2 (median of 87 s) (*p* < 0.001), it recovered to the original level in period 3 (median of 61 s).

**Table 3:** Dispensing time per prescription (second) before and after introducing the robotic dispensing system

	**Period 1 (*n* = 221)**	**Period 2 (*n* = 181)**		**Period 3 (*n* = 310)**		
	**Median (IQR), seconds**	**Median (IQR), seconds**	***p*–values vs Period 1**	**Median (IQR), seconds**	***p*–values vs Period 1**	***p*–values vs Period 2**
Work time						
Pharmacists	60 (26–176)	69 (40–130)	0.364	23 (12–48)	*p* < 0.001*	*p* < 0.001*
Robot	–	19 (12–38)	–	17 (10–32)	–	–
Pharmacy support staff	–	–	–	21 (10–45)	–	–
TOTAL	60 (26–176)	87 (54–167)	*p* < 0.001*	61 (35–140)	0.556	*p* < 0.001*

The work time of robot is defined as the operating time of Drug Station® for each prescription. In period 1, the work time of pharmacists (defined as the time required for manually preparing and dispensing medicines by pharmacist) was measured. In period 2, the work time of robot and the work time of pharmacists (including the total time required for preparing medicines using Drug Station® by one pharmacist and dispensing medicines by another pharmacist) were measured. In period 3, the work time of robot and the work time of pharmacy support staff (defined as the time required for preparing medicines using Drug Station® by pharmacy support staff) and the work time of pharmacists (defined as the time required for dispensing medicines by pharmacist) were measured

*Abbreviation*: *IQR* Interquartile range

^*^ Statistically significant after adjustment using the Bonferroni correction (*p* < 0.0167 for Mann–Whitney *U* test)

## Discussion

Replacing manual dispensing, which requires human resources, with automated dispensing robots is critical for enhancing clinical care for patients by pharmacist. Additionally, collaboration with pharmacy support staff is critical. In this study, we established the robotic dispensing system by using automated dispensing robots and collaborating with pharmacy support staff; subsequently, we evaluated the safety and the efficiency of those systems. The results of this study clearly revealed that the incidences of dispensing errors were significantly reduced immediately after introducing the robotic dispensing system (period 2), and these reduced incidences were maintained after collaboration with pharmacists and pharmacy support staff (period 3). Additionally, the dispensing time of pharmacists was significantly reduced after introducing the system (period 3). To the best of our knowledge, this is the first study that evaluates the safety and efficiency of implementing automated dispensing robots and a collaborative working model with pharmacists and pharmacy support staff in Japan.

The incidence of unprevented dispensing errors in the previous studies was 0.003–0.047% [11, 12]. The incidence of unprevented dispensing errors in this study at baseline (period 1) was 0.015%, which is consistent with previous reports. Thus, the accuracy of dispensing process in our hospital appeared to be within the general level in Japan. Therefore, the results in this study can be generalized to other institutions nationwide.

The incidences of total prevented dispensing errors was significantly reduced from 0.204% in period 1 to 0.054% (a reduction rate of 73.5%) and 0.044% (a reduction rate of 78.4%) in periods 2 and 3, respectively. These results were consistent among wrong quantity, wrong strength, wrong drug, and wrong dosage form. Notably, the number of cases of wrong strength, wrong drug, and wrong dosage form were nearly zero in period 3. The error type of wrong quantity, which was the most frequent type of errors in period 1, was reduced to approximately 25% in period 2, and subsequently the incidence of this type of error was similar in period 3. The most case of wrong quantity in periods 2 and 3 occurred when we used Hp-PORIMS® (data not shown). These results were consistent with those of a previous study [6]. In contrast with Hp-PORIMS®, which requires manual checking of the number of medicines, Drug Station® can confirm the number of medicines using the built-in camera and/or built-in electric scale. We consider that the results of this study reflect the characteristics of each system. The error type of others was not changed after introducing the robotic dispensing system (periods 2 and 3). These cases included human errors, such as putting medicines into wrong labeled paper bag, and these cases should be focused on even when we use robotic dispensing systems.

Similar to prevented dispensing errors, the incidences of total unprevented dispensing errors were significantly reduced from 0.015% in period 1 to 0.005% (a reduction rate of 66.7%) and 0.002% (a reduction rate of 86.7%) in periods 2 and 3, respectively. After introducing the robotic dispensing system (periods 2 and 3), the absence of unprevented dispensing errors related to wrong strength, wrong drug, or wrong dosage form revealed a remarkable safety benefit.

The dispensing process is extremely complicated because pharmacists must select accurate medicines, quantities, strengths, etc., from among more than 2,000 medicines. These are written on each patient's prescription. In addition, dispensing errors can cause serious iatrogenic harm to patients. Therefore, the partial involvement of pharmacy support staff in the dispensing procedure is not accelerated nationwide. Although no original study has been published on collaborative work with pharmacists and pharmacy support staff in Japan, some studies have been published in the world [13–17]. Numerous hospitals face significant shortage of hospital pharmacists, who are necessary for enhancing clinical care for patients in Japan. This issue has been highlighted herein for the first time, with the intention of resolving it. We demonstrated that the robotic dispensing system enabled the medication dispensing duties of the pharmacist to be partially and safely shared with automated dispensing robots and pharmacy support staff in Japan.

To date, numerous automated dispensing devices have been implemented, and safety and efficiency in the dispensing process has been achieved by implementing these devices [2, 4, 5, 13–19]. We introduced the automated dispensing robot (Drug Station®) and the automated dispensing robot for powdered medicines (Mini DimeRo®) [4], the bar-corded dispensing support system (Hp-PORIMS®) [6]. Although the effect of the latter two devices have been reported [4, 6], that of Drug Station® have not been reported. In Japan, press-through package (PTP, also known as blister pack) sheet, bottle, sachet etc. are available as pharmaceutical packaging types of oral or topical medicines. Therefore, a system for preventing dispensing errors and improving the efficiency of dispensing processes for several types of medicines was required. Previously, the effect of implementing the automated dispensing robot, which was only available for PTP sheets of tablets or capsules (robo-pick®, Yuyama Co., Ltd., Osaka, Japan), was reported in Japan [2]. However, Drug Station® can not only store PTP sheets but also other dosage forms (topical medications, etc.) and their various types of pharmaceutical packaging. Additionally, because the automated dispensing robot is equipped with the visual and gravimetric verification of each medicine, we could delegate part of the dispensing process from pharmacists to pharmacy support staff. The results of this study revealed that the incidence of dispensing errors after introducing the robotic dispensing system (period 2) were reduced. Additionally, the reduced rate was maintained after starting collaboration with the pharmacists and pharmacy support staff (period 3). Notably, the number of cases of wrong strength and wrong drug, which can seriously impact a patient's health [20], reduced to almost zero immediately after introducing the robotic dispensing system.

In this study, the work time of pharmacists in medication dispensing was shared with Drug Station® and pharmacy support staff after introducing the robotic dispensing system in period 3. Consequently, the work time of pharmacists in medication dispensing was significantly reduced after introducing the robotic dispensing system. The total dispensing time significantly increased from periods 1 to 2 and recovered to the original level in period 3. Possible causes of these differences included the unexpected behaviors or inexperienced operators of the automated dispensing robot in period 2. Thus, we considered that the effects of the robotic dispensing system for the dispensing time were reflected in period 3.

To summarize, introducing the robotic dispensing system enabled the process of medication dispensing by pharmacist to be partially shared with automated dispensing robots and pharmacy support staff. Additionally, after introducing the robotic dispensing system, the incidences of total prevented or unprevented dispensing errors were reduced by approximately 80% than that before introducing the system (from periods 1 to 3). Among them, the number of cases of wrong strength and wrong drug, which can cause serious iatrogenic harm to patients, were reduced to almost zero, and these results exhibit clinical implications for safe dispensing. The results of the study suggest that the robotic dispensing system by using automated dispensing robots and collaborating with pharmacy support staff is one of the ways to enhance clinical care for patients ensuring quality and safety of medication by pharmacists.

This study had some limitations. First, the prescribed medicines and pharmacists were not exactly the same in each period, because of the patient and staff turnover. Secondly, we performed an uncontrolled before-after study, because dispensing could not be randomized after introducing the automated dispensing robots. Although this method is often used in other studies [13–17, 19], it is inherently susceptible to bias owing to the lack of a control group. Therefore, a quasi-experimental method such as an interrupted time series analysis is needed for incorporating long-term outcomes in future studies.

## Conclusions

The robotic dispensing system enabled the process of medication dispensing by pharmacist to be partially and safely shared with automated dispensing robots and pharmacy support staff. Therefore, clinical care for patients by pharmacist could be enhanced by ensuring quality and safety of medication.

## Supplementary Information

**Additional file 1: Supplementary Table 1.** Characteristics of prescription used for evaluating dispensing time.

## Data Availability

All data generated or analyzed during this study are included in this published article.

## Abbreviations

PDA: Personal digital assistance
CPOE: Computerized physician order entry
P&T Committee: Pharmacy and Therapeutic Committee
PTP: Press-through package
