# Supplementary material for: Evaluating the safety and efficiency of robotic dispensing systems
Source: J Pharm Health Care Sci. 2022 Oct 1;8:24. doi: 10.1186/s40780-022-00255-w (PMC9526262; doi:10.1186/s40780-022-00255-w)
Supplement: Supplementary file 1 — Additional file 1: Supplementary Table 1. Characteristics of prescription used for evaluating dispensing time. [file 40780_2022_255_MOESM1_ESM.docx]

# **Supplementary Table 1** Characteristics of prescription used for evaluating dispensing time

|  | **Period 1** | **Period 2** | **Period 3** |
| --- | --- | --- | --- |
| No. of prescriptions dispensed, n | 223 | 184 | 310 |
| No. of medication orders per prescription dispensed, median (IQR) | 2 (1–5) | 2 (1–4) | 1 (1–3) |
| No. of prescriptions including each package form dispensed, n (%) |  |  |  |
| Single unit package | 201 (90.1%) | 179 (97.3%) | 304 (98.1%) |
| One-dose package | 46 (20.6%) | 32 (17.4%) | 5 (1.6%) |
| Powder | 37 (16.6%) | 26 (14.1%) | 27 (8.7%) |
| Liquids | 2 (0%) | 0 (0%) | 2 (0.6%) |

*Abbreviation*: *IQR* interquartile range
